# A zoonotic focus of cutaneous leishmaniasis in Addis Ababa, Ethiopia

**DOI:** 10.1186/1756-3305-2-60

**Published:** 2009-12-02

**Authors:** Wossenseged Lemma, Girume Erenso, Endalamaw Gadisa, Meshesha Balkew, Teshome Gebre-Michael, Asrat Hailu

**Affiliations:** 1 College of Medicine and Health Sciences, Department of Medical Laboratory Technology, Gondar University, PO Box 196, Gondar, Ethiopia; 2 Armauer Hansen Research Institute (AHRI), PO Box 1005, Addis Ababa, Ethiopia; 3 Aklilu Lemma Institute of Pathobiology (AIPB), Addis Ababa University (AAU), PO Box 1176, Addis Ababa, Ethiopia; 4 Faculty of Medicine, Department of Microbiology, Immunology & Parasitology, (DMIP), Addis Ababa University, PO Box 9086, Addis Ababa, Ethiopia

## Abstract

**Background:**

Cutaneous leishmaniasis (CL) is endemic in the highlands of Ethiopia, and almost always caused by *Leishmania aethiopica*. Hitherto, Addis Ababa (the capital city of Ethiopia) was not considered endemic for CL, mainly due to absence of epidemiological and field ecological studies. This report summarizes the preliminary epidemiological investigation that proved the existence of active transmission in southeastern Addis Ababa.

**Results:**

Active case finding surveys were conducted in 3 localities, Saris, Kality, and Akaki, which are found in and around Bulbula-Akaki river gorges. During the surveys conducted in January 2005 - May 2006, a total of 35 cases with 9 active and 26 healed skin lesions were identified. Eighteen of the cases (51.4%) were found in Saris; while 10 (28.6%) and 7 (20%) cases were from Kality and Akaki respectively.

Ten colonies of rock hyraxes (*Heterohyrax brucei*) were identified in the vicinities of the 3 localities. Three of the 48 hyraxes (6.3%) trapped from the surroundings harbored natural infections of *Leishmania aethiopica*. Confirmation of the *Leishmania *species of the 3 isolates was achieved by PCR amplification and RFLP analysis of the ribosomal DNA internal transcribed spacer (ITS) sequences. Based on sandfly species composition and proximity of resting sites to human settlements, *Phlebotomus longipes *is circumstantially proven to be the vector of CL in south east Addis Ababa.

**Conclusion:**

The study proves the existence of isolated zoonotic foci of CL in south eastern Addis Ababa, with *P. longipes *as the likely vector and *H. brucei *as the natural reservoir host.

## Background

Ethiopian Cutaneous Leishmaniasis (ECL) is a widespread skin disease caused mainly by *Leishmania aethiopica*, but rarely by *L. tropica *and *L. major*; the latter two species cause ECL in the lowland regions [1-3]. The disease presents in three clinical forms: localized cutaneous leishmaniasis (LCL), mucocutaneous (MCL) and diffuse cutaneous leishmaniasis (DCL) [1-4]. LCL lesions are often benign and self healing; occasionally resulting in severe and persistent lesions. Persistent/severe LCL, MCL and DCL lesions are disfiguring [4-6], and often require protracted treatment schedules. In the case of DCL, definite cure is hardly ever achieved, since relapse is common. Precise figures on the numbers of ECL cases are lacking. Based on unofficial estimates, the total number of ECL cases diagnosed each year is around 20,000 [7].

Two species of hyraxes, *Procavia capensis *and *H. brucei*, have been incriminated as reservoir hosts of *L. aethiopica*, with a natural infection rate of 21-27% reported in some rural areas [8]. Two closely related sandfly species, i.e., *Phlebotomus longipes *and *P. pedifer *have also been identified as proven vectors [8,9]. These sandfly species are mainly found in the altitude ranges of 1400 - 2700 m [1,10], thus limiting the distribution of ECL in the Ethiopian highlands [4,10]. However, a lower altitudinal limit of CL (1200 m) has been suspected [5], suggesting a wider altitudinal distribution of *Phlebotomus longipes *and *P. pedifer*, or implicating the vector potential of other species. The recent isolation of *L. aethiopica *from *P. sergenti *in the lowlands of Awash valley [11] and from a ground squirrel (*Xerus rutilus*) in low lying plains of southern Ethiopia [12] suggest a wider altitudinal range of ECL.

Female sandflies of the species *P. longipes *and *P. pedifer *readily feed on hyraxes, and share their habitat [8,13]. Hyraxes also accumulate organic matter in their latrines and create a suitable breeding environment for sandflies [8,10,13]. This intimate ecological association between the two sandfly species (*P. longipes *and *P. pedifer*) and rock hyraxes is characteristic of ECL; and almost always gives a clue on the existence of the disease in any locality, especially in the highlands of Ethiopia [10,14].

Addis Ababa, the capital of Ethiopia, has not been considered by many as a CL endemic focus. Thus, the CL patients diagnosed in the various health facilities of the city were considered by many experts as imported cases. To refute this misconception, and aiming to prove the existence of an active transmission within Addis Ababa, we launched epidemiological, ecological and entomological investigations in a section of the city. We herewith describe the findings, based on the surveys conducted in selected locations in and around the Bulbula-Akaki river gorge that traverses through the city from the center towards the south east.

## Materials and methods

### Study areas

Addis Ababa, being the capital city of Ethiopia, is home to 22.9% of all urban dwellers of the country, with a population size of 2,738,248 in 2007 [15] residing in an estimated area of 530.14 km^2 ^[16]. The city is located on coordinates 9°02'N 38°44'E9.03°N 38.74°E and altitude ranges of 2326 to over 3000 meters [16].

**Figure 1:**
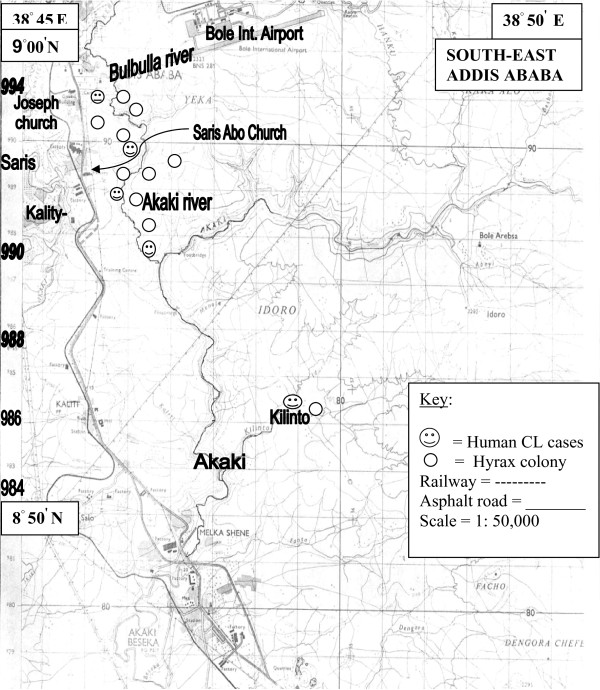
**Map of study areas showing Bulbula - Akaki gorge**.

The three study localities described in this report; namely Saris, Kality and Akaki are found in and around the gorges of Bulbula-Akaki river. Altitude-wise, these localities lie between 2326 and 2500 m (Figure 1). The basalt rock cliffs along the sides of Bulbula-Akaki gorge are covered with shrubs and trees; the commonest trees being species of *Acacia*, *Ficus *and *Eucalyptus*.

### Study design and study populations

This multi-disciplinary epidemiological investigation was carried out for a period of 17 months, from January 2005 to May 2006. Ethical approval for the conduct of the study was granted by the Institutional Ethics Review Board of the Department of Biology, Faculty of Science - Addis Ababa University. The study subjects were individuals with active or healed CL lesions. All patients who participated in the surveys gave informed consent. The skin lesions were often identified by the community in its vernacular name 'Shahign', which often was an accurate diagnosis of CL. A single interviewer visited the houses of CL patients to document demographic variables like date of birth, sex, profession, duration of residence in the study area, travel history, and medical history with respect to skin lesions and any previous treatments sought. The surveys were guided by a community informer and two CL patients. The unique non-pigmented, but mottled and depressed scars of healed lesions were used as operational criteria to diagnose past CL. Papular, nodular or ulcerative lesions were noted and used to make a clinical diagnosis of LCL. Patients with multiple non-ulcerative nodular lesions, often bigger in size from those lesions of LCL patients were identified as DCL. The final diagnosis of LCL and DCL was achieved by parasitological diagnosis as described below. The aim of the study was to document the numbers of past and current cases of CL, and to confirm that the infections were not imported from elsewhere.

### Entomological studies of sandflies

For studies involving sandflies, geographical reference points were set from houses of CL patients. All searches for resting sites of sandflies, and trapping sites were within a 1.0 km radius from the identified houses. We considered this as a reasonable estimate of the flight ranges of *P. longipes *[17]. Sandflies were aspirated directly from their resting sites using a mouth suction apparatus, and captured by CDC light traps (Model512, Hock and Co., USA) set outside or inside houses. The CDC traps were set at 6 p.m. and left overnight till 6 a.m. hanging at about 0.5 meter above ground level. Throughout the study period, 1 trap per site per week was installed in 3 outdoor sites and inside houses of 3 CL patients (in Saris and Kality). Captured sandflies were brought to Aklilu Lemma Institute of Pathobiology (AIPB) for species identification and dissection of females in an attempt to grow *Leishmania *parasites in culture. Possible resting sites of *P. longipes *were searched in residential areas for two days between 6 a.m. and 10 p.m. in order to locate domestic resting sites. Seasonal abundance of *P. longipes *was determined from CDC light trap catches of each month; and expressed as total numbers of *P. longipes *per month. No attempt was made to identify the species of sandflies belonging to the genus *Sergentomyia*.

### Reservoir host studies

The search for animal reservoir hosts and the ecological studies of the same, targeted rock hyraxes and small rodents. Rock hyraxes were captured by locally made and previously tested snare traps placed in the mouths of tree and rock holes, crevices of rocks, and along the walking paths of hyraxes. Rodents were captured using commercially available collapsible traps (Bio Quip product, USA) baited with peanut butter. The trapping of rodents was attempted from within or in close proximity to hyrax and sandfly habitats. A permission to trap hyraxes and rodents was obtained from the Ethiopian Wild Life Conservation & Development Authority. The size and body weights of hyraxes, were used to broadly classify age groups as young, juvenile and adult. Colony sizes of hyraxes were estimated by direct counting of individual hyraxes in peak hours of diurnal activities, i.e. during grazing and sun basking. Repeated counts over a long period revealed more or less accurate numbers.

### Diagnosis of CL and *Leishmania *species identification

Diagnosis of CL lesions involved clinical examination, followed by parasitological procedures. Dermal scrapings of lesions (or intact skin in case of hyraxes) obtained from incised skin slits were smeared on microscope slides and examined for presence/absence of amastigotes after staining with Giemsa. The dermal scrapings were simultaneously inoculated into NNN (Novy MacNeal Nicolle) blood agar base medium overlaid with Locke's solution containing 100 units of penicillin and 100 μg of streptomycin per milliliter. In addition to skin, whole blood samples and biopsies of internal organs (liver, spleen and bone marrow) were obtained and treated in a similar manner. Inoculated NNN culture vials were incubated at room temperature (23 - 26 C°) for a maximum of 3 weeks and examined weekly for growth of promastigote stages. When promastigotes were found in culture, they would be sub-cultured into fresh NNN medium, and also used to infect golden hamsters (*Mesocricetus auratus*). Hamster infections were initiated by inoculation of 5 × 10^6 ^stationary phase promastigotes into the skin, usually in the nose region.

For *Leishmania *species identification, genomic DNA was extracted from a harvest of 10^6 ^stationary phase promastigotes employing the phenol-chloroform-isopropanol extraction and ethanol precipitation methods. LIST (5'-CTGGATCATTTTCCGATG-3') and L5.8S (5'-TACCACTTATCGCACTT-3') primer pair was used for PCR reactions. The PCR products of the isolates from hyraxes along with those of reference strains were separated by electrophoresis on 1.8% agarose gel and analyzed as previously described [18]. The PCR products, once confirmed by the detection of *Leishmania *specific bands, were further digested with HhaI and fractionated by electrophoresis in 2% agarose gel. Species-specific restriction patterns were used to identify the *Leishmania *species [18].

## Results

### Human infections

A total of 35 patients with 9 active and 26 healed skin lesions were detected during the 17-month survey between January 2005 and May 2006. Except for three patients (2 females and 1 male) with DCL lesions, the rest 6 were LCL. 97% of the healed lesions were in the faces of the patients. The majority of patients 18 (51.4%) cases came from Saris; while 10 (28.6%) were from Kality, and the rest 7(20.0%) from Akaki. The age group 0 - 9 and 10 -19 were the most affected (Table 1). Four of the 12 patients with age above 30 were employed as night guards in the local Church and other commercial establishments found in the locality.

**Table 1:** Age and sex distribution of CL patients with active and healed lesions living in the settlements around Bulbula-Akaki gorge (January 2005 - May 2006).

Age group (years)	Males No. (%)	Females No. (%)	Total (%)
0 - 9	5 (14.3)	3 (8.6%)	8(22.9)

10 - 19	4 (11.4)	7 (20%)	11(31.4)

20 - 29	2 (5.7)	2 (5.7%)	4(11.4)

30 - 39	2 (5.7)	4 (11.4)	6(17.1)

40 - 49	1 (2.9)	1(2.9%)	2(5.7)

> 50	3 (8.6)	1(2.9)	4(11.4)

**Total**	**17 (48.6)**	**18 (51.4)**	**35(100)**

### Entomological data on sandflies

**Figure 2:**
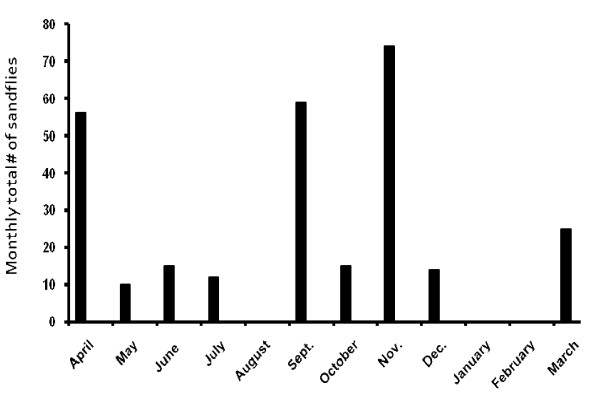
**Monthly sandfly abundance in southeast Addis Ababa, April 2005-March 2006; based on CDC light trap catches**. N.B. Monthly totals are based on the sums of sandfly numbers collected 4-days a month (weekly) in 3 sites, using 1 CDC light trap per site. No sampling was done February 2006.

Using CDC light traps, and by active day time searches in possible resting sites, a total of 1307 phlebotomine sandflies (663 males, 644 females) were collected between April 2005 and March 2006. All the sandflies belonged to the species *P. longipes*. The highest numbers of sandflies were captured in November, followed by September and April (Figure 2). During day time searches, no sandflies were found resting in domestic areas. Nine CDC light traps suspended overnight inside houses of 3 CL patients captured 18 *P. longipes *(17 females and 1 male). *Leishmania *parasites were not found in the guts of 580 female *P. longipes *dissected.

### Animal reservoirs

Hyraxes and rats (*Rattus rattus *and *Praomys *spp) were the predominant peri-domestic animals found in the study areas. A 17 month (January 2005 - May 2006) ecological observation in hyrax habitats revealed the existence of at least 10 colonies, with colony sizes of approximately 12-15 individuals; all belonging to the species *Heterohyrax brucei*. Pregnant hyraxes were found in June, September and November, 2005. Newly born and juvenile hyraxes (0.2 - 0.3 kg) were seen and trapped between September and December 2005. Hyraxes inhabited the crevices and cracks of the basalt rocks on the cliffs of the Bulbula-Akaki river gorge, as well as the hollow openings of the giant *Ficus *trees. As expected, hyraxes came out of their rock or tree holes mostly early in the morning and at dusk. A maximum of 12 hyraxes were seen sunbathing in the mornings.

A total of 51 hyraxes were trapped between January 2005 and May 2006. No sampling was done in January and August. Only two hyraxes were trapped in December and February, while a minimum of 4 hyraxes were trapped in the remaining months of the year. NNN cultures detected promastigotes from skin tissue of 3 of the 48 hyraxes examined (6.3%), while cultures obtained from the rest 3 hyraxes were contaminated. The three *Leishmania *isolates were found among hyraxes trapped from Saris (2 out of 18; 11.1%) and Kality (1 out of 17; 5.9%); 2 juveniles and 1 sub-adult. Microscopic examination of Giemsa stained smears of skin, blood and visceral organs obtained from all 51 hyraxes did not reveal amastgote stages. No visible skin lesions were present in both infected and uninfected hyraxes.

Experimental infections were initiated in 16 hamsters using the 3 isolates of hyraxes; and all except one were successfully infected. Consistent with our previous *L. aethiopica *infection experiments in hamsters (unpublished observations), the lesions were nodular in nature, and did not form ulcers up to 1 year after evolution.

None of 14 *Rattus rattus *and the 12 *Praomys *sp. had skin lesions; and none were found infected.

### Species identification by PCR/RFLP

**Figure 3:**
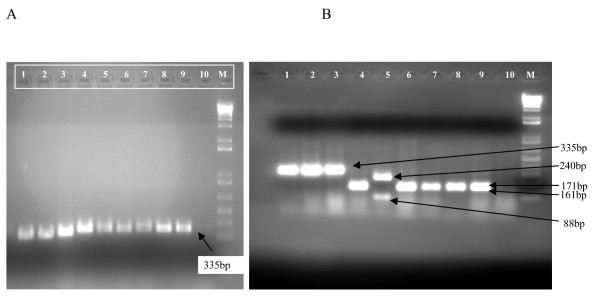
**ITS-1 PCR product (graph A) and restriction patterns of *Leishmania *ITS-1. PCR product digested with *Hhal *(graph B)**. Lanes: 1 = *L. infantum *(MHOM/FR/LEM-75); 2 = *L. tropica *(MHOM/SU/74/K27); **3 = *L. donovani ***(MHOM/IN/80/DD8); 4 = *L*. *aethiopica ref *(MHOM/ET/72/L100); 5 = *L. major *(MHOM/SU/73/5-ASKH); Lanes 6 - 9 are isolates from hyraxes: 6 = H11/SK, 7 = H2/SK, 8 = H32/SK, 9 = H02/SK; 10 = Negative control; M = 100 bp molecular marker ladder. N.B. H02 and H2 originate from a single hyrax; H02 had been passaged in hamster infection, re-isolated and processed by PCR/RFLP analysis.

The PCR product of ITS-1 region amplified with primer sets LIST and L5.8S gave approximately a 335 bp band on agarose gel electrophoresis for the 3 *Leishmania *strains isolated from hyraxes (H02/H2, H11 and H32) and the reference strains of: *L. major (MHOM/SU/73/5-ASKH)*, *L. aethiopica *(MHOM/ET/72/L100), *L. tropica (MHOM/SU/74/K27)*, *L. donovani *(MHOM/IN/80/DD8) and *L. infantum *(MHOM/FR/LEM-75) (Figure 3).

HhaI digests of the amplified ITS-1 region, upon agarose gel electrophoresis, generated a restriction pattern yielding 2 poorly resolving bands of 161 and 171 bp in all the 3 isolates similar to those of a reference strain of *L. aethiopica *(MHOM/ET/72/L100). Other bands of approximately 88 and 240 bp were produced in *L. major *reference strain; while a single band of 335 bp was produced in *L. donovani*, *L. tropica *and *L. infantum *reference strains (Figure 3).

## Discussion

In this study, in which we aimed to prove the existence of local transmission in Addis Ababa, the true prevalence of CL could not be determined due to unavailability of demographic data. Based on numbers of active cases, it can be inferred that the point prevalence during the surveys was less than 5 per 10,000 persons. The majority (54.3%) of the patients were children and young adults; both sexes being equally affected; 48.6% in males versus 51.4% in females (p < 0.05). As this interpretation includes patients with past lesions, the prevalence in children is probably higher than we found. Human behavior has been considered as one of the important factors affecting the age and sex distribution of CL in different foci [8,10,19,20]. The preponderance of the disease in males has been reported repeatedly both in health facility [6] and community-based field studies [4,19]. For instance, the male to female ratio of CL patients diagnosed at All African Leprosy Rehabilitation and Training centre (ALERT) from May 1981 to April 1983 was 1.6:1, even though this could well be a consequence of the gender discrepancy in the health seeking pattern [6]. In this study, we have observed a more or less equal distribution of the disease in males and females, probably reflecting the low rates of transmission, and/or due to its preponderance in children. Reminiscent of this, Wilkins [19] reported a low prevalence of CL in Meta Abo (a locality 25 kms west of Addis Ababa), and also showed that both males and females were equally affected. In a highly endemic area of south western Ethiopia, males and females of age under 10 were equally affected [20].

We observed that houses with CL cases were found closer to the gorge and hyrax colonies than houses without cases. Furthermore, children and young adults would frequently visit the gorges during day time for leisure and recreation or for fetching water. Many individuals swam or took a bath in the meager stream of water flowing in the gorge, mainly during the rainy season. Others would go to the church, which is also located at the escarpment of the gorge, spending many hours in spiritual ceremonies held in the evenings and mornings. However, not many individuals would stay in the gorge during night time. Merely based on these observations, it is hardly possible to point out where and at which time of the day sandflies bite humans. Detailed accounts of entomological risk factors, which were beyond the scope of this study, are needed to answer these questions.

Taking note of the proportionally large numbers of patients with past lesions (74.3%; 26 out of 35), it can be inferred that CL transmission in the Bulbula-Kality gorge and its environs has been taking place for at least 3 decades, albeit with low incidence. Thus our finding of CL in Addis Ababa is a discovery of an existing problem. However, what remains to be determined is whether or not there has been an increasing trend in the numbers of cases over the past years; and to confirm if indeed man-to-man transmission takes place.

The only wild mammals, aside from hyraxes, that were found in large numbers around the residential quarters of CL patients were *Rattus rattus *and *Praomys spp*. However, none were found to be infected. In laboratory experiments, these rodents were not the preferred sources of blood meal for *P. longipes *[4,17], and were not found naturally infected with *L. aethiopica *[4,8,17]. Baboons (*Papio anubis*) and monkeys (*Cercopithecus aethiops*) that could readily be infected with *L. aethiopica *in the laboratory [21] were not found in the study area. On the contrary, *H. brucei *was found throughout the gorges of Bulbula-Kality, with at least 10 colonies identified in the study areas. However, the colony sizes were relatively small as previously noted [8].

In Figure 2, we show that peak numbers of *P. longipes *were found in the months September through November. This perfectly coincides with the main *H. brucei *breeding season that we have noted. We were able to detect natural infections of *Leishmania *in 2 juvenile and 1 sub-adult hyraxes in April, May, and early September 2005. Age and season dependent *Leishmania *infections have previously been demonstrated in Tunisia for *L. major *natural infections of *Psammomys obesus *[22]. In Ethiopia, previously reported natural infection rates of *Leishmania *in hyraxes were in the ranges of 3.5% - 27%, showing seasonal variation in rates of natural infections [8]. The months when natural infection in hyraxes would be found were May in Kutaber (NE Ethiopia); July in Ochollo (SW Ethiopia); and March, May and July in Aleku (Western Ethiopia). In Kutaber, the lowest natural infection rate of 3.5% was found among 84 hyraxes trapped between April 1971 and April 1972 [8]. This data on seasonality of natural *L. aethiopica *infection in hyraxes highlights two important questions, i.e., 1) its impact on seasonality of infection in man, and 2) longevity (life span of patency) of the natural infection in hyraxes. As it stands now, we have no proof that the higher infection rate of juvenile hyraxes is not due to chance or sampling bias. Experience shows that younger animals are usually naïve and daring; hence being vulnerable to predators and assault by enemies. Similarly, younger hyraxes compared to their older counterparts, are easily trapped. These observations warrant a critical re-assessment of the seasonality of natural infections of rock hyraxes by *L. aethiopica*.

Unlike *L. tropica*, which produce lesions in golden hamsters within 4 weeks [23], the *Leishmania *strains isolated from hyraxes produced lesions only after 18 weeks, and did not ulcerate for 1 year after evolution. This observation corroborates previous *L. aethiopica *infection experiments involving golden hamsters [24] and baboons (*Papio anubis *and *P. gelada*), but contrasts with the infection outcome in grivet monkeys [21] and humans. This hamster infection experiment using the 3 *Leishmania *isolates from hyraxes confirms the well known behavior of the parasite. The isolation of *Leishmania aethiopica *from *H. brucei*, and the peculiar outcomes of the infection in hamsters are consistent with previous knowledge; and clearly signify the important role that hyraxes play as reservoir hosts of CL in Addis Ababa. Further, the identification of *P. longipes *as the predominant phlebotomine sandfly closely associated with hyrax colonies and human habitations emphasizes the zoonotic nature of the disease. Previous reports have documented the existence of *P. longipes *in Addis Ababa [4,25], and shown the seasonal fluctuations of its population [8,17]; notably, a summer and winter decline and an autumn and spring peak abundance. Our inability to find infected *P. longipes *appears worrying given the fact that natural infections of this species are not rare. Infection rates of 1.6% in Meta Abo [25] and 3.7% in Kutaber [8] have been reported. The detection of natural infection in sandflies by conventional parasitological methods remains to be a challenge. Future studies are expected to rely on PCR based techniques to enhance the sensitivity of detection. We found that 65% of the dissected *P. longipes *females were nulliparous, which partly explains why no infections were found.

The increasing number of CL cases in south east Addis Ababa is here confirmed to be an outcome of an ongoing transmission of *L. aethiopica *in isolated zoonotic microfoci. Due to the expansion of the city, areas previously considered rural have become urban; as a result of which, humans have intruded into the habitats of rock hyraxes (*Heterohyrax brucei*), concurrently becoming victims of CL. In the long run, hyraxes can be expected to disappear from the area due to urbanization and industrialization; however, the disease can linger and become anthroponotic. The extent to which man-to-man transmission contributes to the burden of CL in Addis Ababa needs to be determined, so as to devise a control strategy appropriate to the metropolitan setting.

This study confirms the existence of local transmission of CL in southeastern parts of Addis Ababa, and provides preliminary data on the possible roles of *P. longipes *and *H. brucei*. The data, albeit limited in scope, further highlights the zoonotic nature of the disease in Addis Ababa. Future studies will address trends in prevalence, and assess the risks of man-to-man transmission in a city which is expanding its frontiers.

## Competing interests

The authors declare that they have no competing interests.

## Authors' contributions

LW, GT, EG and HA designed the study. LW, EG, GT and HA organized the field studies. LW and GE conducted the molecular works. LW, BM and GT organized field and laboratory works involving sandflies. GT and HA supervised the overall conduct of the study. WL, EG and HA looked after patients' needs, i.e., diagnostics and treatments. LW and HA prepared the draft manuscript. All authors read the manuscript. LW and HA are the guarantors of the manuscript.
